# Supplementary material for: Dietary magnesium intakes among women of reproductive age in Ghana—A comparison of two dietary analysis programs
Source: PLoS One. 2023 May 2;18(5):e0284648. doi: 10.1371/journal.pone.0284648 (PMC10153694; doi:10.1371/journal.pone.0284648)
Supplement: Attachment — Submitted filename: Response to comments_18Feb23.docx [file pone.0284648.s003.docx]

February 25, 2023

We want to thank the editor and reviewer for their comments and suggestions. We have made the suggested changes and believe that the manuscript is strengthened. We have tracked the changes we made in the manuscript and provided responses to each of the comments below.

**Response to editor’s comments**

When submitting your revision, we need you to address these additional requirements.

1. Please ensure that your manuscript meets PLOS ONE's style requirements, including those for file naming. The PLOS ONE style templates can be found at

https://journals.plos.org/plosone/s/file?id=wjVg/PLOSOne_formatting_sample_main_body.pdf and

https://journals.plos.org/plosone/s/file?id=ba62/PLOSOne_formatting_sample_title_authors_affiliations.pdf

We have ensured our manuscript meets PLOS ONE’s style requirements

2. Please include a complete copy of PLOS’ questionnaire on inclusivity in global research in your revised manuscript. Our policy for research in this area aims to improve transparency in the reporting of research performed outside of researchers’ own country or community. The policy applies to researchers who have travelled to a different country to conduct research, research with Indigenous populations or their lands, and research on cultural artefacts. The questionnaire can also be requested at the journal’s discretion for any other submissions, even if these conditions are not met. Please find more information on the policy and a link to download a blank copy of the questionnaire here: https://journals.plos.org/plosone/s/best-practices-in-research-reporting. Please upload a completed version of your questionnaire as Supporting Information when you resubmit your manuscript.

We have completed the questionnaire.

3. Thank you for stating the following financial disclosure:

"Supported by startup funding awarded to Brietta Oaks from the University of Rhode Island, USA"

At this time, please address the following queries:

a) Please clarify the sources of funding (financial or material support) for your study. List the grants or organizations that supported your study, including funding received from your institution.

No other grants or organizations supported this study.

b) State what role the funders took in the study. If the funders had no role in your study, please state: “The funders had no role in study design, data collection and analysis, decision to publish, or preparation of the manuscript.”

The funders had no role in study design, data collection and analysis, decision to publish, or preparation of the manuscript.

c) If any authors received a salary from any of your funders, please state which authors and which funders.

None of the authors received funding for this specific research project.

d) If you did not receive any funding for this study, please state: “The authors received no specific funding for this work.”

The authors received no specific funding for this work.

4. We note that you have indicated that data from this study are available upon request. PLOS only allows data to be available upon request if there are legal or ethical restrictions on sharing data publicly. For more information on unacceptable data access restrictions, please see http://journals.plos.org/plosone/s/data-availability#loc-unacceptable-data-access-restrictions.

In your revised cover letter, please address the following prompts:

a) If there are ethical or legal restrictions on sharing a de-identified data set, please explain them in detail (e.g., data contain potentially sensitive information, data are owned by a third-party organization, etc.) and who has imposed them (e.g., an ethics committee). Please also provide contact information for a data access committee, ethics committee, or other institutional body to which data requests may be sent.

There are no ethical or legal restrictions.

b) If there are no restrictions, please upload the minimal anonymized data set necessary to replicate your study findings as either Supporting Information files or to a stable, public repository and provide us with the relevant URLs, DOIs, or accession numbers. For a list of acceptable repositories, please see http://journals.plos.org/plosone/s/data-availability#loc-recommended-repositories.

We have now uploaded a minimal anonymized data set.

5. Please amend either the title on the online submission form (via Edit Submission) or the title in the manuscript so that they are identical.

We have amended the title on the online submission form to match the title in the manuscript.

6. Please ensure that you refer to Figure 2 in your text as, if accepted, production will need this reference to link the reader to the figure.

We have cited Figure 2 in the manuscript.

**Response to Reviewer’s comments**

Major comments

This is the article studying daily magnesium intake using different two methods of FFQ. The authors showed that one of the methods seems better to that of another. In ither words, ESHA seems superior to NDSR because ESHA involves West African Food database for magnesium. However, my concern is that in the methods part, the authors state that the magnesium content of specific Ghanaian foods not available in either ESHA or NDSR were obtained from the Food and Agriculture Organization West African Food Composition table (lines 146 – 8). From this description, magnesium content could be obtained both to ESHA and NDSR. If so, if the responder responded food A which is not involved both, the authors could obtain magnesium content to both and there might not be different between ESHA and NDSR.

We thank the reviewer for this thoughtful comment. ESHA's database contains some foods that are not present in the West African food composition table, therefore even though we supplemented both tables as needed with the West African food composition table, ESHA came out on top because it contained items that were not found in the composition table. Lines 237-240 provide this explanation. We kept the practice of supplementing both databases with West African food composition tables because that is how they are used in practice.

The other small concerns are as the follows:

1. the readers could not understand which foods are related with magnesium intake. In the other words, there are no descriptions which food are more frequently taken to explain to magnesium intake are more. To explain this, the authors must show the relationship of most of daily magnesium intakes as nutrient with food which had taken most.

This information can be found in Figure 2. We provided further information in the discussion which can be found in Lines 213-217.

2. No information of frequencies of foods intakes to explain magnesium intake.

Our dietary data was based on a 7-day food frequency questionnaire, therefore the frequency of food is not discussed further, as the frequency (weekly) is built into the questionnaire.

3. The authors showed that daily magnesium intake using ESHA and NDSR were significantly different, but as far as the figure 1 and standard deviation (SD) of both described in the article in lines 179 and 180, the difference between the two is not seen significant different because of large SDs of them.

Thank you for your comment. We have corrected this problem by presenting the means with SEs rather than SDs, and we have updated Figure 1. Since this is a paired sample comparison, we have included the mean difference, SE, and 95% CI, which does not contain zero and indicates that the mean difference between the groups is statistically significant (Lines 179-181)

4. No information of pregnancy outcomes with magnesium deficiency is described because the outcome of magnesium deficiency must be critical issue in this article. For resolve this, the authors must show the relationship between magnesium deficiency and pregnancy adverse outcomes using the objective outcome parameters.

While we agree that this is an important topic to investigate, it is outside the scope of this manuscript and study because we did not collect data on birth outcomes.

Minor comments

1. The tables to show the relationships between daily intake of foods and magnesium to explain which foods are most to contribute to magnesium deficiencies.

This information is provided in Figure 2.
